# Gamified Practice Improves Paretic Arm Motor Behavior in Individuals With Stroke

**DOI:** 10.1177/15459683241286449

**Published:** 2024-09-28

**Authors:** Cristina Rubino, Bimal Lakhani, Beverley C. Larssen, Sarah N. Kraeutner, Justin W. Andrushko, Michael R. Borich, Lara A. Boyd

**Affiliations:** 1 Graduate Program in Rehabilitation Sciences, University of British Columbia, Vancouver, BC, Canada; 2 Department of Physical Therapy, University of British Columbia, Vancouver, BC, Canada; 3 Department of Psychology, University of British Columbia, Okanagan, BC, Canada; 4 Department of Sport, Exercise and Rehabilitation, Faculty of Health and Life Sciences, Northumbria University, Newcastle upon Tyne, UK; 5 Division of Physical Therapy, Department of Rehabilitation Medicine, Emory University, Atlanta, GA, USA

**Keywords:** stroke, motor learning, motor function, gamified rehabilitation

## Abstract

**Background:**

Stroke is a heterogeneous condition, making choice of treatment, and determination of how to structure rehabilitation outcomes diﬃcult. Individualized goal-directed and repetitive physical practice is an important determinant of motor learning. Yet, many investigations of motor learning after stroke deliver task practice without consideration of individual capability of the learner.

**Objective:**

We developed a gamified arm rehabilitation task for people with stroke that is personalized to individual capacity for paretic arm movement, provides a high dose of practice, progresses through increasingly difficulty levels that are dependent on the performance of the individual, and is practiced in an engaging environment. The objectives of the current study were to determine if 10 days of gamified, object intercept training using the paretic arm would improve arm movement speed and clinical outcome measures of impairment or function.

**Methods:**

Individuals with chronic stroke and age-matched controls engaged in 10 days of gamified, skilled motor practice of a semi-immersive virtual reality-based intercept and release task. The paretic arm was assessed using the Fugl-Meyer Assessment (motor impairment) and Wolf Motor Function Test (motor function) before and after training.

**Results:**

Both groups showed faster arm movement speed with practice; individuals with stroke demonstrated reduced paretic arm motor impairment and increased function after the intervention. Age and sex (for both groups), and time post-stroke were not related to changes in movement speed.

**Conclusions:**

Findings indicate that gamified motor training positively affects paretic arm motor behavior in individuals with mild to severe chronic stroke.

## Introduction

There is a 1 in 4 lifetime risk of a stroke after age 25 years.^1^ Owing to significant advances in acute medical interventions, stroke is now considered a chronic disease in much of the world; for example, 83% of Canadians will survive at least 1 year following a first stroke.^2,3^ However, most stroke survivors suffer from long-term functional disability that significantly affects their quality of life.^4^ Further complicating recovery, stroke is a heterogeneous condition, making the choice of treatment and the determination of how to structure rehabilitation outcomes diﬃcult. Despite this, clinical care is often designed with a “one size ﬁts all” point of view, which can make it vulnerable to patient heterogeneity, poor therapeutic responses, and lead to incomplete recovery.

Numerous studies have demonstrated significant levels of inactivity following stroke.^5,6^ This problem is often compounded by the low amounts of practice delivered during stroke rehabilitation.^6^ The amount of paretic arm use in daily life after stroke is also extremely low as compared to matched controls. Using accelerometry, Lang et al^5^ noted that healthy controls use their upper extremities 8 to 9 hours per day. In contrast individuals with stroke use their paretic and non-paretic upper extremities substantially less, 3.3 and 6.0 hours per day, respectively.

Goal-directed, task-specific, and repetitive physical practice is an important determinant of motor learning in animal studies,^7,8^ human motor skill learning,^9-11^ and the reacquisition of motor skills during neurorehabilitation.^12,13^ Beyond the quantity of practice required for motor learning, there are important considerations about the quality of practice. Not all practice may be equally efficacious for learning,^14,15^ which is defined by long-term retention.^16^ Most investigations of motor learning after stroke deliver a task for practice without consideration of the individual capability of the learner.^17-19^ Yet personalizing practice to an optimal difficulty level for each participant would ensure that task demands do not exceed, or fall below, cognitive and motor abilities.^14^ Guadagnoli and Lee^14^ proposed the challenge point framework as a conceptual approach to manipulating task demands to meet each individual’s capacity. In theory, matching motor capacity with task demands maximizes the potential for behavioral change associated with motor learning.

In addition, many lab- or clinically-based motor learning interventions rely on repetitive task practice in the absence of an engaging environment.^20-22^ Yet neurophysiological evidence suggests that motivation and engagement can affect learning. Rodent research shows that “enriched” environments (those containing complex inanimate and social stimulation) can increase the retention of new neurons,^23^ the number of synapses per neuron,^24^ and together with task-specific reaching therapy, augment recovery of forelimb motor function poststroke.^25^ Complimenting these findings other work shows that training in an engaging, game environment improves the learning of a novel motor skill compared to an equal amount of mechanically similar training in a less engaging environment.^26^ Thus, it may be important to develop motor interventions that stimulate a high dose of practice,^27^ personalize difficulty of movement,^14,15,19^ and provide an engaging and motivating environment.^26^

To address these needs, we developed a gamified arm rehabilitation task for individuals with stroke, the Track And Intercept Task (TrAIT).^28^ TrAIT was designed to: (1) be personalized to individual capacity for stroke-affected arm movement, (2) provide a high dose of practice, (3) increase in difficulty as skill is gained, and (4) be practiced in an engaging, gamified environment.

Thus, the objectives of the current study were to determine if 10 days of gamified, object-intercept training (TrAIT) using the paretic arm in a personalized workspace would alter: (1) arm movement speed in the training task, and (2) clinical outcome measures of motor impairment and function in individuals with chronic stroke. To determine who may be best positioned to benefit from our gamified rehabilitation task, we tested whether baseline clinical scores correlated with the magnitude of behavioral change. We hypothesized that gamified task practice would lead to: (1) faster paretic arm movement speed, and (2) reduced paretic arm motor impairment or increased function.

## Methods

### Participants

Forty individuals with chronic stroke and 30 healthy older adults were recruited for this study. To ensure that changes in arm motor impairment or function were attributable to gamified task practice, we tested individuals in the chronic stage after stroke (>6 months post-stroke). Individuals with stroke and healthy controls were between the ages of 35 to 85 years. Individuals from both groups were excluded if they: (1) showed signs of cognitive impairment (<24 on the Montreal Cognitive Assessment,^29^ (2) could not engage in the motor task without arm or shoulder pain, or (3) had history of head trauma, seizure, psychiatric diagnosis, neurodegenerative disorder, substance abuse, or other neurological or muscular deficits that affected vision or manual control. All testing was conducted at the University of British Columbia. Informed consent from each participant was obtained according to the Declaration of Helsinki. The Research Ethics Board at the University of British Columbia approved all study procedures.

### Clinical Assessment

Trained physical therapists rated paretic arm motor impairment (Fugl-Meyer upper-extremity scale [FM-UE])^30^ and function (Wolf Motor Function Test [WMFT])^31^ 24 hours before and after 10-sessions of TrAIT practice. The FM-UE is a measure of upper extremity impairment and is rated on a scale from 0 to 66, with higher scores reflecting less impairment.^30^ The WMFT consists of 15 timed items. WMFT rate (repetitions/60 seconds) was calculated for each item to increase sensitivity to detect change in individuals with moderate to severe functional impairment; equation (1).^32^ If an individual was not able to complete 1 repetition of a task within 120 seconds, a task rate of zero was assigned. Overall WMFT rate was calculated as the average of the rates of the 15-time based functional tasks.

(1) $$\mathrm{Task}\mathrm{rate}=\left(\begin{array}{l} 60\;\left(\mathrm{seconds}\right)\\ /\mathrm{Performance}\mathrm{time}\left(\mathrm{seconds}\right) \end{array}\right)$$

### Behavioral Task

A semi-immersive virtual reality-based intercept and release task, called TrAIT, was designed to optimally challenge each individual as it was tailored to each individual’s movement capabilities.^28,33,34^ TrAIT was performed using an apparatus consisting of a 46″ monitor with a Microsoft Kinect camera (model no. 1517, Kinect for Windows; Microsoft, Redmond, WA, USA), positioned 72 inches away from the participant. Immediately prior to each session, the task was calibrated to each individual’s arm active range of motion by asking participants to reach as far as they could to all 4 corners of the screen. Individual calibration ensured that all participants could perform the reaching task, customized the workspace, and allowed for adjustments to the task workspace as participants gained improved arm mobility across practice sessions. The task required participants to move their paretic (stroke), or non-dominant (healthy control), arm to control an on-screen icon (spaceship) to intercept moving objects (asteroids). Once the object was intercepted, the participant “threw” it toward a target (the sun). The physical requirements of the task (intercept and throw) simulated discrete waving-like motions (eg, cleaning a window). Failing to intercept the asteroid caused it to fall toward the bottom of the screen and explode. The location of the sun randomly varied (top, bottom, left, and right) on the screen. Auditory cues (ie, sound effects) and visual feedback, along with a cumulative score reflecting successful asteroid intercept and throw, were continuously employed to maintain motivation and engagement throughout the task.^26,28^

Ten TrAIT practice sessions were completed by each participant, resulting in 2 to 3 practice sessions per week for 4 weeks. Each session lasted approximately 30 minutes and consisted of 5 consecutive TrAIT blocks with breaks provided between blocks. Each block contained 200 movements (5 blocks each with 100 object interceptions and 100 object throws) for a total of 1000 paretic or non-dominant arm movements performed per session. A total of 10 000 arm movements were performed in the experiment; 5000 object interceptions and 5000 object throws. The paretic arm was not supported during the task which was purposely designed to include and retain participants with a wide range of motor functional abilities. Participants could perform the task using a closed fist and some trunk movement if required. Since the task required discrete whole-arm movements, participants who had difficulty maintaining a raised arm throughout practice could perform 1 trial, rest their arm on their lap, and then raise their arm again for the next trial. The task became progressively more difficult as skill was gained. Twelve levels were pre-programmed, each associated with increasing difficulty. Individuals advanced to the next level after they achieved 80% accuracy (intercepting and throwing asteroids) on 2 consecutive blocks of practice. Difficulty levels were increased by manipulating object speeds and sizes of objects and targets. The size of the target (sun) and object (asteroid) decreased every 3 levels. Within each set of 3 levels, the velocity deviation increased from 0 to 1.38 units/frame, resulting in an increase in the average velocity of all presented asteroids within a level, starting at 83 pixel/sound in level 1 and reaching up to 500% relative to level 1. The size of the sun decreased after levels 3, 5, 7, 9, and 10 to a minimum size of 20% relative to level 1. Asteroid size also decreased every third level to a minimum size of 40% relative to level 1. Time constraints for both interception and throwing were introduced at higher levels, starting from level 7, ranging from no limits to a maximum of 1 second of total time. To advance to the next difficulty level, participants had to achieve a minimum score of 80% (defined as successful object interception and hitting the target) on 2 consecutive blocks of practice.

Changes in task difficulty associated with success allowed each individual to perform in a personalized environment that contained optimal challenges, based on the principles of the challenge point framework.^14^ An optimally challenging environment was created in several ways. First, the workspace was customized to require that individuals reach to 80% of their capacity. This was measured and adjusted every session so that as reaching ability improved, the workspace was enlarged. Second, task difficulty was progressed based on individual performance. While each participant started at level 1 of difficulty, as soon as they performed 2 blocks in a row with 80% of asteroids caught and thrown, they automatically progressed to the next level. Given this control, each participant progressed at their own rate.

**Figure 1.**
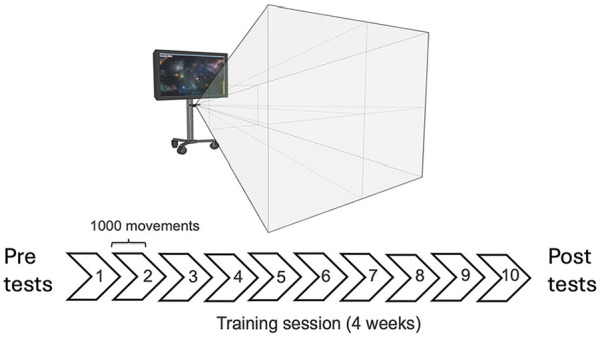
Participants engaged in 10 sessions of a gamified sensorimotor reaching task over 4 weeks, amounting to 10 000 total arm movements. Participants used their affected (stroke) or non-dominant arm (control). Clinical scores were measured 24-hour before (pre) and after (post) training.

### Data Analyses

Data were obtained using custom code and behavioral measures were extracted using custom MATLAB scripts. Movement time was obtained for each trial. To quantify movement time (MT), all trials in which participants successfully intercepted the object and accurately threw it to the target were first identified. Then, MT was calculated as the time between trial onset and object interception, for all successful trials. A missed object or incorrect throw was calculated as an error and not included in the final analyses. A motor skill acquisition change score was calculated as mean MT at training day 10 minus training day 1, for each participant. Motor-learning-related change is characterized by fitting behavioral data to an exponential equation.^35^

(2) $$E\left(MT_{N}\right)\;=\;A\;+Be^{-\alpha N}$$

E(MT) is the expected value of **MT** on trial **N**. The **B** score (seconds) is calculated from learning curves and is a measure of overall change in MT from the beginning of training to the point when performance change plateaus. Secondary outcome measures **A** (seconds) identifies the MT at which the participant has plateaued in performance and **α** (seconds/trial) quantifies the rate of skill acquisition to the point of plateau.^15,19,33^

### Statistical Analysis

Statistical analyses were performed in RStudio (v.4.2.0). Data normality was assessed using Shapiro–Wilk tests, and visualized using *QQ* plots, boxplots, and histograms. Non-parametric statistical testing was conducted if statistical assumptions of parametric tests were violated. To test for motor skill acquisition, mean MT between training day 1 and day 10 was examined using paired tests within each group. Correlations tested the relationship between mean MT change score and age for control and stroke participants, and time post stroke for stroke participants. Two-samples *t*-test assessed the difference of MT change score by sex for each group. To test whether there was a relationship between motor skill acquisition and clinical outcome measures, correlations were performed for acquisition curve parameters (*B*, α) and clinical scores (WMFT rate, FM-UE). Further, *t*-tests were performed to assess the difference in clinical outcome measures at the post- compared to pre-training timepoint. Additionally, we explored our data to determine whether FM-UE change scores differed for distal and proximal subscales of the FM-UE. We assessed subscales of the FM-UE using the following criteria:^36,37^ (1) Item’s 2, 3, 4, 5, and 9 were summed and represented proximal (shoulder/arm) function, (2) item’s 7 and 8 were summed and represented distal (wrist/hand) function, and (3) item’s 1 and 6, which assess reflexes, were removed. Distal and proximal subscales were then grouped based on the amount of total FM-UE change observed. That is, individuals were grouped into 1 of 2 categories: (1) changed FM-UE by 4 or more points, or (2) changed FM-UE less than 4 points.

## Results

Ten of 40 individuals with stroke did not complete the study: 4 were excluded due to ineligible criteria (stroke lesions outside of MCA; confirmed after enrollment), 4 were no longer interested or had scheduling conflicts, and 2 dropped out due to being too impaired to perform the task (assessed on training day 1; both FM-UE = 8). Three of 30 controls did not complete the study: as the study required MRI scans (reported separately) 2 were excluded due to incidental findings and 1 dropped out due to arm fatigue (after completing ~50% of training sessions). Thirty individuals with stroke and 27 controls completed the study. Of those who completed the study, 4 individuals with stroke and 3 controls were excluded from final analyses for incomplete data sets, leaving 26 stroke (66 ± 11 years old, 9 females, 58 ± 45 months since stroke) and 24 controls (65 ± 8 years old, 17 females; Table 1).

**Table 1.** Demographic, Movement Time, and Clinical Data for Participants.

Participant	Group	Sex	Age (y)	Trained arm	Pre mean MT (s)	Post mean MT (s)	Mean MT change (s, pre-post)	Stroke hemisphere	Time since stroke (mo)	Pre FM-UE score	FM-UE change (post-pre)	WMFT rate (reps/min) change (post-pre)
S1	Stroke	F	47	L	3.47	4.06	−0.58	R	35	10	1	4.56
S2	Stroke	F	37	R	3.65	3.35	0.30	L	84	18	5	−0.43
S3	Stroke	M	51	L	3.38	2.68	0.70	R	14	23	9	0.45
S4	Stroke	M	61	R	1.95	1.31	0.65	L	62	28	1	−3.38
S5	Stroke	F	51	L	2.14	1.13	1.01	R	47	29	0	−2.39
S6	Stroke	M	60	L	2.35	1.30	1.05	R	188	31	1	2.27
S7	Stroke	F	57	R	4.19	1.47	2.73	L	162	32	0	−1.88
S8	Stroke	M	59	R	3.27	2.17	1.11	L	61	33	−3	6.32
S9	Stroke	F	74	L	2.86	2.00	0.86	R	19	39	8	−3.58
S10	Stroke	M	72	L	1.75	0.89	0.86	R	49	50	4	11.30
S11	Stroke	M	69	R	2.43	1.34	1.09	L	66	51	3	12.36
S12	Stroke	F	71	R	1.82	1.03	0.79	L	94	52	12	11.54
S13	Stroke	M	73	R	2.28	0.98	1.30	L	60	54	4	7.43
S14	Stroke	M	79	L	2.36	1.46	0.90	R	27	54	0	9.17
S15	Stroke	M	77	R	2.06	1.35	0.71	L	32	58	5	−6.89
S16	Stroke	M	58	R	2.34	0.97	1.37	L	96	59	−2	−1.82
S17	Stroke	M	62	L	1.80	0.95	0.85	R	8	59	5	4.40
S18	Stroke	M	71	L	1.69	0.95	0.74	R	117	59	0	−5.41
S19	Stroke	F	79	L	1.80	1.18	0.62	R	51	59	6	−1.32
S20	Stroke	M	62	R	1.90	1.03	0.87	L	6	62	−1	3.68
S21	Stroke	M	80	L	1.82	0.96	0.86	R	40	62	−2	4.69
S22	Stroke	F	70	L	1.93	1.28	0.65	R	76	64	1	9.07
S23	Stroke	M	73	L	1.62	1.15	0.47	R	47	64	−2	0.11
S24	Stroke	F	78	L	2.24	1.26	0.98	R	28	64	−1	−25.25
S25	Stroke	M	75	R	1.58	0.78	0.80	L	41	65	−4	9.78
S26	Stroke	M	66	L	1.57	0.96	0.61	R	10	66	−4	55.50
C1	Control	F	47	L	1.62	0.88	0.74					
C2	Control	F	50	L	1.45	0.75	0.71					
C3	Control	F	56	L	1.95	0.79	1.16					
C4	Control	F	58	L	2.39	1.00	1.39					
C5	Control	F	58	L	1.69	0.88	0.81					
C6	Control	F	58	R	1.85	0.79	1.06					
C7	Control	M	58	L	1.37	0.86	0.51					
C8	Control	F	61	L	1.83	0.97	0.86					
C9	Control	F	62	L	1.70	0.96	0.74					
C10	Control	F	62	R	2.33	0.94	1.39					
C11	Control	F	63	L	1.60	0.90	0.69					
C12	Control	F	63	L	1.45	0.71	0.74					
C13	Control	M	63	L	1.92	0.83	1.10					
C14	Control	F	65	L	2.03	1.18	0.85					
C15	Control	F	67	L	1.53	1.21	0.32					
C16	Control	M	67	L	1.70	0.97	0.73					
C17	Control	M	68	L	1.74	0.81	0.93					
C18	Control	M	71	L	1.53	0.98	0.55					
C19	Control	F	73	L	2.43	0.95	1.48					
C20	Control	F	73	L	1.66	0.84	0.81					
C21	Control	F	74	L	1.51	0.94	0.57					
C22	Control	M	75	L	1.31	0.81	0.50					
C23	Control	F	77	L	2.14	1.20	0.94					
C24	Control	M	81	L	1.61	0.95	0.66					

The remaining participants in both groups completed 10 000 movements across 10 practice days without mention of adverse effects. Participants were prospectively matched for age between groups, and a 2-samples Wilcoxon test confirmed that age was not different between groups (*P* = .35).

### Motor Skill Acquisition After 10 days of Skilled Arm Practice

**Figure 2.**
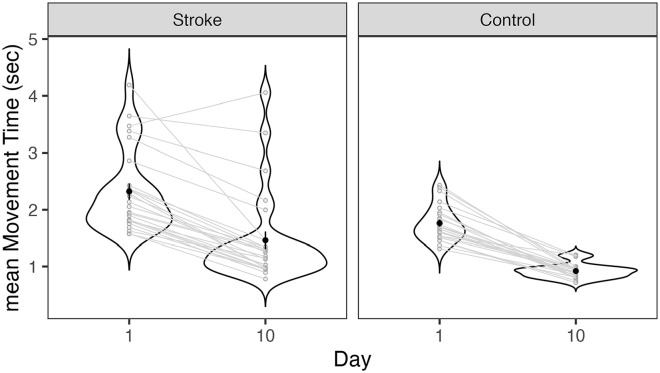
Movement time mean (seconds; MT) of initial (day 1) and final (day 10) training days for control and stroke participants. Individual gray lines represent paired data for each participant. Black dots represent MT mean (averaged for each group and time-point) and vertical lines represent standard error of the mean. There is a significant difference in median MT at day 10 compared to day 1 for each group.

An average of 84.2 ± 7.9% (stroke) and 85.9 ± 5.6% (controls) of the asteroids were successfully intercepted during training. On average, the final level of TrAIT achieved was 7 ± 3 (range: 1-11) for individuals with stroke and 9 ± 2 (range: 6-12) for controls. On average, over all training sessions, it took participants 25 ± 8 min to complete each session (~5 min/block). Participants improved range of motion of their trained, paretic arm (maximum area of the screen that could be reached; as assessed during calibration, prior to each testing session) by 12.7%. MT change scores (day 10 minus day 1) were evaluated for normality, and 2 stroke participants were detected as outliers (Shapiro–Wilk test, *P* < .05). MT at day 1 and day 10 were also tested for homogeneity of variance, and the control group did not pass Levene’s Test (*P* < .05). Thus, non-parametric tests testing the difference of day 10 versus day 1 were performed for each group. Wilcoxon signed rank tests of paired data (day 1 vs day 10) revealed a significant difference in median MT at day 10 compared to day 1 for both stroke (*Z*(26) = −5.25, *P* < .001, effect size *r* = .86, median difference = 0.88 seconds) and control (*Z*(24) = −5.29, *P* < .001, effect size *r* = .88, median difference = 0.77 seconds) participants (Figure 2).

**Figure 3.**
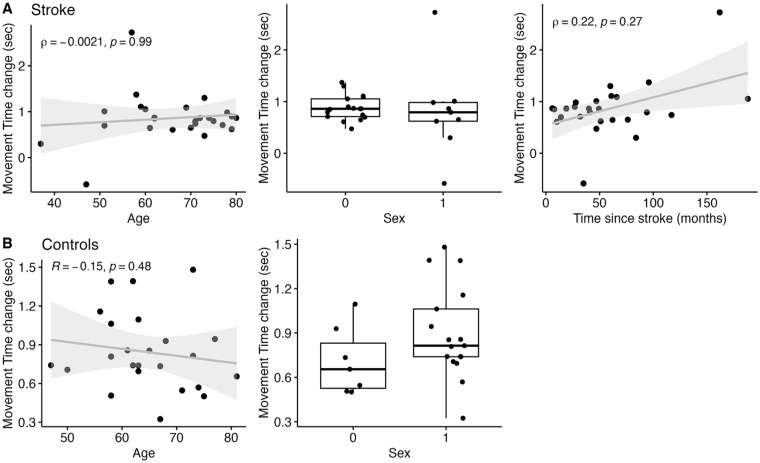
Correlations between demographic variables (age, sex, and time since stroke) and MT change for stroke (A) and control (B) participants. Age and sex (both groups), and time post-stroke were not related to changes in movement speed.

The relationship between MT change score (mean MT day 10 minus mean MT day 1) and age was assessed using correlations and revealed no significant relationship for controls (*r* = −.15, 95% CI [−0.52, 0.27], *t*(22) = −0.72, *P* = .48) nor stroke participants (ρ = −.002, *P* = .992; Figure 3). The relationship between MT change and time since stroke was assessed using a correlation, which revealed no significant relationship (ρ = .22, *P* = .272). Further, unpaired *t*-tests of MT change based on sex revealed no significant difference for controls (mean difference = −0.19, 95% CI [−0.46, 0.08], *t*(22) = −1.45, *P* = .160; Cohen’s *d* = −0.62) nor stroke participants (*Z*(24) = −0.742, *P* = .458, effect size *r* = .153, median difference = 0.068 seconds).

Overall, these findings show motor skill was acquired, as assessed by task-specific MT, for all participants. Further, MT change was not affected by age, sex, or time since stroke.

### Clinical Outcome Measures Associated With Motor Skill Acquisition

**Figure 4.**
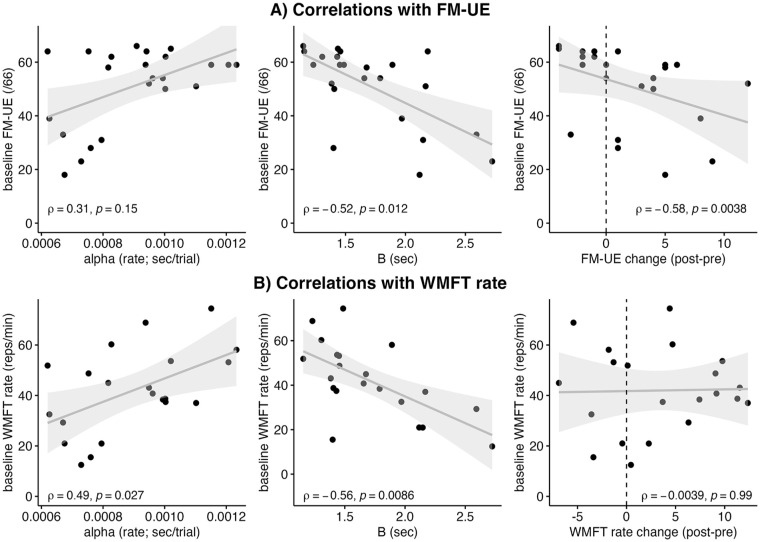
Correlations between skill acquisition curve parameters (alpha, *B*) and clinical outcome measures (A) FM-UE, (B) WMFT rate (affected arm) in stroke participants. Lower baseline FM-UE score was associated with: greater *B* score, and greater FM-UE change score. Slower baseline WMFT rate was associated with: slower alpha (trial-by-trial training rate), and greater *B* score.

The relationship between baseline clinical scores (FM-UE, WMFT rate) and: (1) changes in clinical scores (post minus pre-training), and (2) parameters of the skill acquisition curve (rate [α], overall change [*B*]) was assessed using Spearman’s correlations (Figure 4). For baseline FM-UE score, a significant relationship was found with *B* (ρ = −.57, *P* = .003) and FM-UE change (ρ = −0.51, *P* = .009), but no significant relationship was observed with α (ρ = .14, *P* = .519). Lower baseline FM-UE scores were associated with higher *B* scores and greater FM-UE change scores. Three outliers were excluded due to extreme *B* and α values (>3SD).

For baseline WMFT rate, a significant relationship was found with *B* (ρ = −.56, *P* = .009) and α (*r* = .53, *P* = .014), but no significant relationship was observed with WMFT rate change (*r* = .02, *P* = .918). Slower baseline WMFT rates were associated with slower α (slower improvement per trial) and higher *B* scores. Two additional outliers were excluded due to extremely large WMFT values (>3SD).

Collectively, these findings suggest that both FM-UE and WMFT baseline scores are related to changes in skilled motor performance, and indicate that baseline motor function and impairment may help inform capacity for change in the TrAIT task.

**Figure 5.**
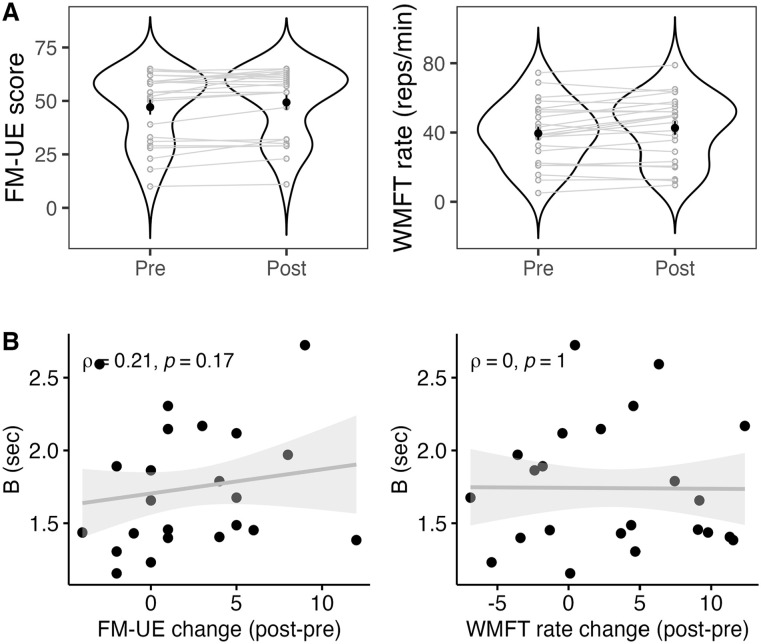
(A) Change in clinical outcome measures from pre- to post-training, with participants in gray and black dot representing mean of each time point. (B) Correlations between skill acquisition curve parameter (*B* score) and changes in clinical outcomes (post minus pre-training) in stroke participants. FM-UE score and WMFT rate increased from pre- to post-training. Change scores for FM-UE and WMFT rate were not associated with *B* score.

**Figure 6.**
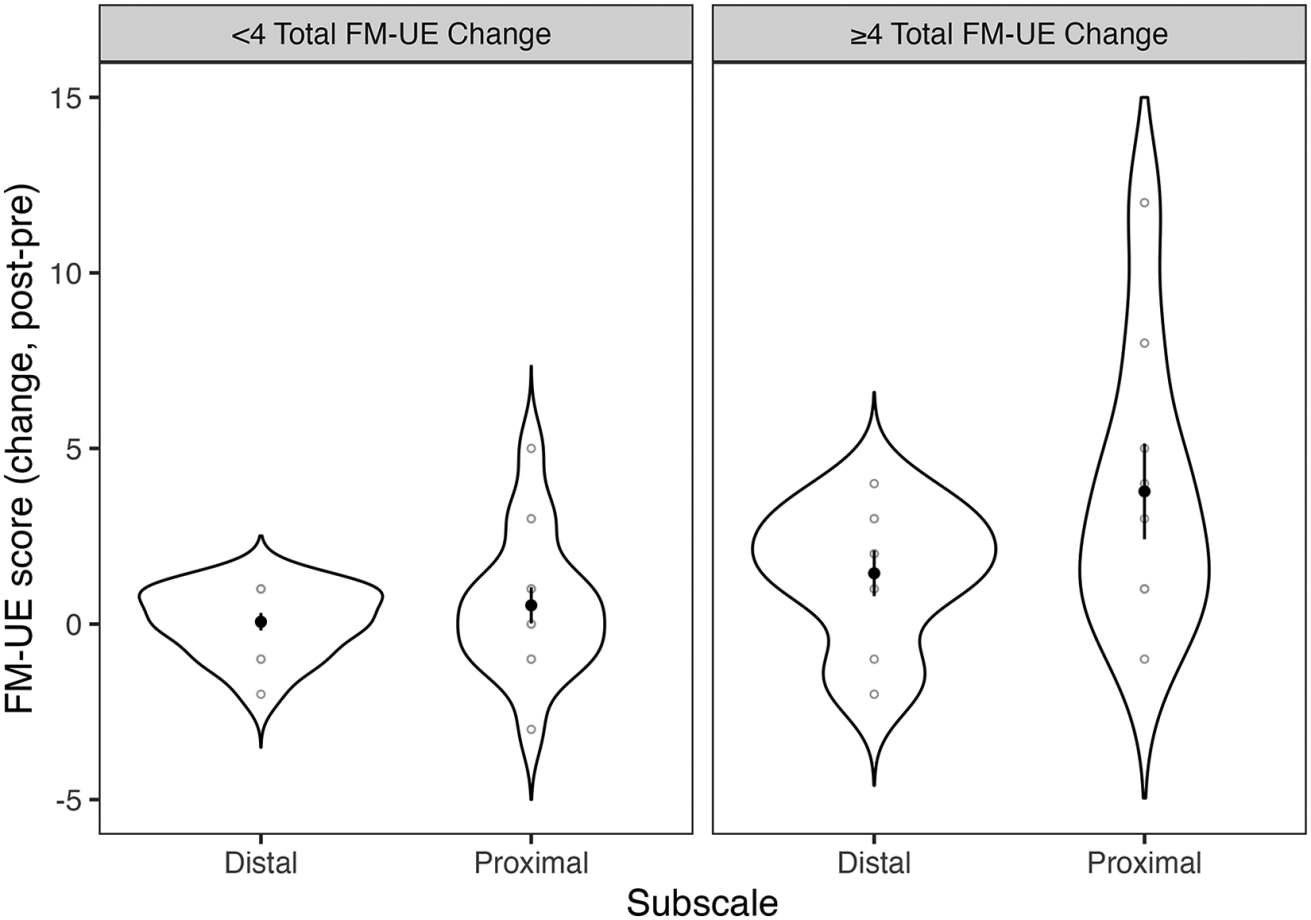
Change scores (post-pre training) for FM-UE divided by group (FM-UE change < 4 versus FM-UE change ≥4) and FM-UE subscale (distal vs proximal). Change scores for each participant are shown in gray open circles. Means are shown in black solid circles, with standard error of the mean as black lines. Participants with FM-UE change score of 4 or more points improved on both the distal and proximal subscales of the FM-UE; there was no significant difference between subscales.

Further, we examined whether clinical scores were statistically different at post- compared to pre-training, and whether change was related to acquisition curve parameter *B* (Figure 5). Paired *t*-test’s showed that FM-UE score significantly increased after training (mean group difference = 2.22 points, 95% CI [0.44, 3.99], *t*(22) = 2.59, *P* = .017; Cohen’s *d* = 0.55). Paired *t*-test’s showed that WMFT rate significantly increased after training (mean group difference = 3.13 reps/minutes, 95% CI [0.62, 5.63], *t*(22) = 2.59, *P* = .017; Cohen’s *d* = 0.55). There was no significant relationship between *B* score and FM-UE change score (ρ = .21, *P* = .169) or WMFT rate change (ρ = .00, *P* > .999). A 2-way mixed analysis of variance was performed to evaluate the effect of FM-UE subscale (distal vs proximal) by group (FM-UE change <4 vs FM-UE change ≥4) on FM-UE change score. There was a significant main effect of group (*F*(1,22) = 12.88, *P* = .002, η_p_^2^ = .37; Figure 6). There was no significant main effect of subscale (*F*(1,22) = 3.74, *P* = .066, η_p_^2^ = .15) or interaction effect.

## Discussion

The current study showed that a large dose (n = 10 000 movements) of skilled paretic arm movement in a gamified environment over 10 days can be accomplished in individuals with chronic stroke and a wide range of initial motor impairment and improves arm movement speed. Further, clinical outcome measures of motor function and impairment were improved after training. Consistent with our hypotheses, we observed behavioral improvements in both stroke and control participants after they engaged in gamified track-and-intercept task training. Faster, more precise arm movements were made by individuals with stroke regardless of age, sex, time since stroke, or pre-training clinical measures. In addition, motor impairment (FM-UE score) and motor function (WMFT rate) improved after training; this change was not dependent on age, sex, or time since stroke. Importantly, these improvements were noted in individuals across a wide range of initial motor impairment after stroke. Taken together, we provide objective evidence that gamified motor training results in faster response of task execution in individuals with chronic stroke across a wide range of initial motor impairment.

### Training-Related Changes in Paretic Arm Movements

Despite the potential benefit of gamified rehabilitation for promoting motor learning and recovery after stroke,^27,38^ many tasks cannot be completed by individuals with severe motor impairment (<30 FM-UE).^39^ While improvements in performing functional motor tasks (measured using WMFT) following gamified practice were demonstrated in previous literature,^40^ gamified practice in individuals across a wide range of initial motor impairment and specifically in individuals with more severe motor impairment is scarce. The TrAIT task was purposefully designed to support inclusion and retention of individuals with a wide range of motor impairment and functional capacity after stroke. This was illustrated in the current study as all participants but one showing improved paretic arm motor behavior indexed by faster task-specific MTs following 10 sessions of training despite a high degree of individual variability in baseline motor impairment (FM-UE range 10-65). Two individuals with severe motor impairment and function (FM-UE = 8; WMFT rate <8 reps/min) had insufficient voluntary arm control to be able to lift their hand into the camera’s field of view to be able to successfully complete the task. Of the 5 participants with severe motor impairment (<30 FM-UE) who completed the study, 4 improved task-specific paretic arm movement speed. One participant showed slower arm movement speed on day 10 compared to day 1. For this participant it is possible that slower arm movement was the result of increased range of motion. Despite having severe motor impairment (FM-UE = 10) and low functional capacity (WMFT rate = 5 reps/minutes), this individual doubled their range of arm motion during reaching on day 10 versus day 1 (20% greater than group average), which could explain their improved arm motor function score after TrAIT (WMFT rate change = 4.5 reps/minutes).

As training progressed, participants were required to make faster paretic arm movements to intercept a moving virtual object and then throw it to a target. Given the multisensory dynamics of the task, behavioral improvement may be the result of improved sensorimotor integration and motor execution in brain regions that support these actions.^33,41^ Past work showed that practice intercepting objects, both real and virtual, led to changes in the structure of white matter in the intraparietal sulcus.^28,42^ For example, Scholz et al^42^ used diffusion tensor imaging to study white matter plasticity associated with learning to juggle in young healthy controls. These authors discovered increases in fractional anisotropy in the right intraparietal sulcus that were only noted in the juggling group and persisted for 4-weeks following training. Similarly, other work suggests that changes in patterns of functional MRI support the learning of complex visuomotor tasks in both healthy participants^43^ and individuals with stroke.^18,33^ It is not clear why change in normalized movement speed (*B* score) was not related to change in clinical scores in the current study. However, our data suggest that all individuals, regardless of magnitude of change in movement speed have the capacity to improve clinical scores. We observed that FM-UE increased by at least 1 point in 14 participants, and by at least 4 points in 9 participants. Although we did not test for minimal detectable change in the current study, an increase in FM-UE by ~4 points has been shown to be a minimal clinically important difference (MCID) in chronic stroke.^44^ Additionally, our data demonstrate that those individuals who showed an increase in FM-UE by at least 4 points improved on both the distal and proximal items of the FM-UE compared to individuals who did not show as much improvement (FM-UE change <4). This suggests that both hand/wrist and shoulder/arm functions as assessed using FM-UE may improve following TrAIT practice. Further, baseline clinical measures did relate to *B* score, demonstrating that individuals with more moderate to severe motor impairment and function show the greatest task-related change. It is likely that individuals with more mild motor impairment showed less task-related change due to fast baseline movement speeds (pre-training mean MT ~1 seconds vs ~2.5 seconds for moderate-severe stroke). It is noteworthy that we trained a wide range of individuals with highly varied ages (37-80 years old) and times since stroke (6-188 months); these factors were not related to changes in movement speed, suggesting that capacity for behavioral change in the paretic limb is not dictated by age or time post-stroke for the chronic phase (>6 months post-stroke).^45^

### Gamified Rehabilitation

Several features of our experimental task may have been key to promoting faster paretic arm movements and reducing paretic arm motor impairment. First, TrAIT delivered a relatively high dose of movements in a short time frame. Individuals completed 1000 additional arm movements each day (500 intercepts, 500 throws) and made a total of 10 000 total movements across the study. Importantly, these movements were additional to those made during daily life, and were completed without any reports of side effects such as shoulder pain. Key to this success was the daily customization of the TrAIT workspace to each individual. Every participant moved to 80% of their available range of motion; as movement capacity increased the workspace was enlarged. The need for an increased number of repetitions during neurorehabilitation has been a subject of debate, with models suggesting that a greater number of repetitions would enhance recovery after stroke,^27^ yet clinical trials have failed to show a benefit of simply increasing the dose of interventions.^20,46^ It is possible that the benefits of TrAIT noted in our study population resulted from the combination of large numbers of repetitions with the performance-specific challenge imposed by scaling the workspace daily to the participant’s available active range of motion. Given that our data are in a chronic population and that the current study was small, future work will have to confirm whether this is indeed the case.

A second feature of TrAIT that may have contributed to our positive findings was its ability to engage participants via the gamified platform. Past work from our group showed that greater engagement as a result of graphics, music, and the reporting of outcomes in a game format led to enhanced motor learning in young healthy controls. In this study we discovered that increased engagement through esthetic, sensory features of the task can facilitate motor learning.^26^ Anecdotally, these features seemed to be at play in the current work, with participants actively engaged in practice sessions and competing with themselves to increase their score of “lives saved” day by day. It appears that gamified tasks can facilitate changes in motor behavior and may impact brain function. Neuroimaging studies in humans implicate dopaminergic midbrain structures (such as substantia nigra and ventral tegmental area)^47-49^ in motivation-mediated learning effects. Changes in these substrates could explain engagement-mediated learning effects; future work will need to confirm this idea.

The TrAIT task was purposefully designed to employ concepts from the challenge point framework.^14^ The challenge point framework describes motor learning–related changes in behavior.^14^ The theoretical point at which an individual reaches a maximum potential for motor learning during practice is known as the “optimal challenge point.” The challenge point reflects an interaction between the skill level of the individual and the unique demands of the task. Theoretically, individualizing practice to an optimal challenge point ensures that task processing demands do not exceed, or fall below, cognitive and motor abilities.^14^ In turn manipulating the task so that it meets each individual’s optimal challenge point would enhance capacity for change associated with motor learning. To date, the notion of individualized optimal challenge points has been considered at the conceptual level,^50,51^ in few studies of healthy controls,^15^ and in case studies of individuals with stroke.^52^ Here we attempted to employ these ideas to maximize behavioral change by linking progression through TrAIT with success. TrAIT contains 10 difficulty levels and requires increased arm movement speed, reaction time and precision targeting in order to progress. Individuals had to show 80% success on 2 successive blocks in order to advance to the next difficulty level. By linking success with difficulty level, we ensured that the task was not too hard or too easy for any individual.

Finally, it is important to note that we did not see a relationship between time since stroke and change in motor behavior suggesting that capacity for improvement remains long after initial injury. In the current study, 10 individuals with stroke showed an increase in FM of at least 3 points. While most of these 10 individuals had relatively mild motor impairment, 2 individuals with severe motor impairment (<30 FM-UE) showed reduced motor impairment. Given the benefits noted with our task our data suggest that gamified interventions that stress functional movements could be useful for enhancing arm motor recovery.

### Limitations

Several factors may limit the application of our findings. Ours was a relatively small study and while we employed a healthy control group, we did not include a group of individuals with stroke who practiced a non-gamified version of our task. We considered that it would be very difficult to recruit and maintain a group of individuals with stroke to complete the same high dose of practice without engagement and personalization of the workspace. While our group of individuals with stroke seems to be very heterogeneous in terms of age, time since stroke, and severity of motor impairment, this diversity allowed us to test whether these factors significantly affected response to TrAIT. While we report improvement in FM-UE test scores we do not know if these changes are greater than the minimal detectable change for FM-UE in the current study sample, thus future study should consider double baseline testing of clinical scales. Moreover, the average change in FM-UE did not reach the MCID, as previously reported in the literature. Nevertheless, given that this study served as an initial assessment of participants’ ability to undergo 10 sessions of TrAIT, future investigations should explore the potential necessity of extending training beyond this threshold to achieve the MCID for all participants. Further, we do know whether task-based improvements in arm use observed in the current study translate to arm use outside of the laboratory. While we did not include an objective assessment of arm use outside of the laboratory, all participants increased arm use through the task itself. Nonetheless, future study should consider how arm use translates to real-world activities such as measured using accelerometry. Finally, as we designed this as a study of motor behavior and an initial test of how the principle from the challenge point framework might be applied in a gamified task for individuals with stroke, we did not include long-term assessments of motor impairment or function. This omission should be corrected in future work that considers whether the impact of gamified rehabilitation tasks, including TrAIT, is maintained over time.

## Conclusions

The current study showed that a gamified rehabilitation task can be tolerated in high doses and minimizes arm motor impairment and increases function in individuals with chronic stroke. We provide evidence that the TrAIT task improves motor behavior regardless of baseline arm motor function and impairment. Our findings support the hypothesis that increasing paretic arm use via the TrAIT task may reduce motor dysfunction and impairment in the chronic stage of stroke recovery. Collectivity, our data suggest that gamified motor rehabilitation interventions may benefit motor behavior after stroke.
